# Restoration of Abnormal Mouths by Surical Treatment before Inserting Plates*Read before the Northeastern Dental Association, at its annual meeting, Worcester, Mass., September 26 to 28, 1917, and reprinted from *Dental Cosmos* for May.

**Published:** 1918-05

**Authors:** James P. Ruyl


### RESTORATION OF ABNORMAL MOUTHS BY SURICAL TREATMENT BEFORE INSERTING PLATES *

BY JAMES P. RUYL, D.D.S.

Among the many things which come to our notice in connection with daily practice are certain abnormalities which occur in the anterior teeth in their relation to the upper and lower jaws. Our interest, as well as a desire for making corrections, has always been excited by them, but they have baffled our efforts for many years. How often do we find mouths with partial dentures replacing the lost bicuspids and molars where the anterior upper or lower teeth—in most cases the uppers—stand out of position, with consequent protruding lips, because of faulty occlusion, and how many times have we seen dentures worn which constantly moved while the wearer was talking! Again, patients come to us who, for want of artificial dentures, show their gums during ordinary lip movement, and when we are called upon to make plates for them we wish they had never come to our office, or we send them to the other man, so that when relatives or friends inquire as to who made their plates, the blame may not rest on us. We have all noticed these unsightly mouths as we go through the streets or sit in front of them in street cars, and while we realized that something might be done to make these people presentable or even good looking, we inwardly hoped that nothing similar would come to us for correction.

Perhaps just as well here as anywhere a far reaching ethical point may be mentioned. I refer to the embarrassment and sensitiveness which abnormalities of any kind

*Read before the Northeastern Dental Association, at its annual meeting, Worcester, Mass., September 26 to 28, 1917, and reprinted from *Dental Cosmos* for May.

bring about. Doubtless all of us have noticed how people with bodily defects have held back, painfully conscious, from mingling with others; how the happiness and freedom of spirit and even the progress of the individual has been impaired. I have known patients to cover an unsightly mouth with their hand and even refrain from smiling, and I myself have rejected an otherwise competent applicant for work because of some dental defect.

Now, for convenience I shall divide the conditions which arise from these abnormalities into four general groups. It is my intention to state them with their causes, and to give an effectual method of correction.

The first and most common one we meet, and one of the most difficult to rectify unless properly handled, is that condition which is brought about because the patient has only six or eight anterior natural teeth, bicuspids and molars having been lost, and has worn an upper denture for many years. In his endeavor to reach for food while chewing, these natural teeth have gradually become loosened and elongated, and there has been a marked forward movment of the process and the teeth, with a consequent thickening of the lower lip, and the appearance of a protruding jaw, often resulting in extreme cases of pyorrhea.

In the mouths of such patients great absorption takes place in the anterior portion of the upper jaw, caused by constant hammering of the lower teeth upon the upper, the upper receding to such an extent that they can not be seen, giving one the impression that there are no teeth in the upper jaw at all. If one wished to make an upper denture for a patient having such conditions in the lower jaw as stated, it would be impossible to construct one that would be satisfactory, because of the lower teeth having become elongated to such an extent that they extend above the line of the occlusal plane—often as much as an eighth of an inch. If we set the centrals and laterals of the upper denture their proper length—about one-sixteenth of an inch below the upper lip line—we should create a condition in which the patient could not close his lips. Now, if we allowed the lips to meet, and did not make a correction in the lower jaw by removing the lower teeth and process, we should have to set the upper teeth so high that there would be an appearance of no teeth in the upper jaw. The result would be a sagging of the mouth, changing what may have been an animated and pliant face into one with a set stern and aged expression, and the new plate would be no better than the old one. My point is that without removal of the teeth and an operation we could not relieve the abnormality.

Next there is the abnormality due to a natural condition where the gums are long and the lips are short. People with such mouths are generally discouraged with their appearance, conscious of their defect, and, being hopeless of any improvement, are prone to neglect their teeth even to the necessary fillings. They lose their teeth early, and the dentist, in making a denture, fits the teeth to the gums, usually with no rubber against the ridge. The teeth must be ground right up to the gum, and in a short time there is an unsightly space between the gums and the teeth. Such a denture is usually very difficult to keep in position and get any degree of comfort in mastication.

The third abnormal condition, due to adenoids, thumb sucking, and irregularities, is a pointed arch with the teeth slanting forward. Now, when such patients come to us there is no longer any possibility of regulation, usually because there are too many teeth lost, or the patient is too old and does not care to undergo treatment for correction of the irregularity.

The fourth abnormal condition is one in which dentures have been worn for many years, and in which the process has been entirely resorbed, leaving a soft flabby ridge. When it falls to the lot of the dentist to make a denture for a mouth of this kind, he, to make a good fit, puts an extremely deep air chamber in the plate, which causes the mucous membrane to pull down and form a cauliflower-like appearance of the tissue, and because of constantly increased flabbiness, not only of the ridge, now, but also of the palatal portion, there is more movement of the plate than before.

All these four conditions must be treated surgically. To leave an unsightly condition, or to augment it, as most dentures will in an abnormal mouth, is not doing justice to a patient. As much of the superfluous process must be removed as will in the judgment of the operator restore a natural comeliness, and in many cases an improvement over nature. Even after the removal of a tooth, in cases where one does not expect to make a facial restoration, it would be advantageous both to the dentist and the patient if, at the time of extraction, the sharp pieces of process that remain where the cervical border of the tooth was were removed. This is often the case with the canine, where the process is a little thicker and more prominent. By so doing we not only hasten the time when we can take the impression, but also save the patient many hours of pain. The sharp prominences that are left by not removing the process are troublesome in eating, etc., and the thin membrane that stretches over it is only a slight protection to the process underneath, and makes it consequently very susceptible to pain.

Those cases with very soft ridges and no process underneath, the process being entirely resorbed, must be treated surgically also, by removing the tissues until there is just a thin portion of the membrane over the bone. By so doing we place the mouth in a condition where the plate rests on a solid foundation, thus preventing its shifting, which would not be the case if the soft tissues were left. It is the same in principle as trying to build a house on a foundation of sand. The operation both for hard and soft tissue is so simple, so easily done—only an obstruction to remove—that it would be more natural to do it than not.

### TECHNIC OF THE SURGICAL PROCEDURE

With the exception of that condition where soft membrane is present, all abnormalities caused by bony prominences are treated in the same way. Novocain or a general anesthetic may be used; I prefer the former. If it is necessary to operate in both jaws make the injections in the upper and lower at once, and after loosening the gum around the teeth with a lancet, extract the teeth only in one jaw and remove the process. When that is finished remove the teeth from the other jaw, first loosening the gum around them as before, and remove the process. My reason for working in this way is to avoid the sponging of blood in places where you are not operating. After removing the teeth, place a blunt instrument, about a quarter of an inch wide, shaped somewhat like an ordinary wax spatula, between the process and the gum, and by leverage, using the process as a fulcrum, tear the mucoperiosteum away, leaving the bone perfectly clean. This is easily done, far more easily than dissecting the gum, and causing much less bleeding. Now hold the gum-flap back, and using a bone forceps or, better still, a curved wedge cutter, cut off as much process as necessary. The sharp edges which are left palatally and labially must be rounded off with a bur or engine stone; apply water with a syringe while doing so.

We now have the flaps of gum hanging down anteriorly and posteriorly, and they can be very easily cut off with gum scissors. Remove enough gum tissue so that the parts will not quite meet when pressed together over the process with the finger and thumb, leaving a space of about one-sixteenth to one-eighth of an inch. By thus not allowing the membrane to come together absolutely, it will be found that when the healing takes place the gum will reach over the bone and leave a firm healthy ridge. It is not necessary to use sutures at any time. Absolute healing takes place and in from a month to six weeks permanent dentures can be made. I have taken impressions for temporary plates in from five to ten days after such operations, and they were worn for from one to two years. In the operation for cases where there is a pointed arch it is generally necessary only to remove the outer plate of process in order to get the lip back to normal. One need not cut off the whole process—only the anterior portion—because in these cases the gums do not show so much, and we only want lip restoration.

For the operation upon soft flabby ridges the anesthetic injection is made in the same manner as before. Instead of beginning at one side of the ridge and cutting entirely across to the other side, begin in the center of the soft tissue and cut on one side almost to the end, leaving only enough attachment to keep the flap from dropping; then begin from the center again, and cut entirely across to the other side. The flap will not be suspended by a small piece, which can easily be clipped off. By doing this we prevent the flaps from getting in the way while operating. In these cases healing takes place so rapidly that impressions can be taken within a week or ten days. The cauliflower-like soft tissues, caused by extremely deep air chambers, are removed in the same manner, except that a curved lancet is used. The tissues should be shaved down to the periosteum.

Such radical changes as this operation makes in the facial expression very often bring us into difficulties with the patient's family, because they have been so accustomed to the old expression that, when a correction is made, even though the expression be markedly improved, it is often very hard to reconcile them to the alteration. However, if a proper denture producing adequate restoration is worn for a week or so, the change for the better is so quickly noticed by the rest of the family that if the old plate were put in again they would not be pleased with it.

It seems to me that this work has brought me more satisfaction than any I have ever done. To overcome difficulties which have usually been neglected, and to bring to a successful issue what was formerly a bugbear, should of course give one great pleasure, but more to me than all this is the joy I have felt in seeing my patients so well satisfied, and the knowledge that I have given them more than mere mechanical service.

